# Spinal Cord Stimulation as an Alternative to Opioid for Axial Neck and Back Pain: A Case Series

**DOI:** 10.3389/fpain.2022.847504

**Published:** 2022-03-08

**Authors:** Graeme Sampson Mullins, Joanna Jane Burns, Andre Perillier Schneider, Antonios El Helou

**Affiliations:** ^1^Faculty of Medicine, Dalhousie University, Halifax Regional Municipality, Halifax, NS, Canada; ^2^Department of Family Medicine, The Moncton Hospital, Moncton, NB, Canada; ^3^Department of Pharmacy, The Moncton Hospital, Moncton, NB, Canada; ^4^Université de Sherbrooke, Sherbrooke, QC, Canada; ^5^Department of Anesthesia and Pain Medicine, Vitalité Health Network, Bathurst Hospital, Bathurst, NB, Canada; ^6^Department of Neurosurgery, The Moncton Hospital, Moncton, NB, Canada

**Keywords:** spinal cord stimulation (SCS), persistent spinal pain syndrome, pain control, opioid reduction, improved quality of life

## Abstract

**Introduction:**

Spinal cord stimulation is emerging as a minimally invasive technique for treatment of persistent spinal pain syndrome (PSPS).

**Methods:**

We describe a case series of 25 individuals with PSPS who underwent implantation of a spinal cord stimulator device between 2017 and 2021.

**Results:**

There was a significant reduction in mean visual analog scale pain scores in the immediate postoperative phase, (8.61 vs. 2.3, *p* < 0.001). There were twelve patients who consumed pre-operative opioid, and 75% showed reduction of use with a significantly lower average daily dose (66.8 vs. 26.9 meq/D, *p* < 0.05). There was a significant reduction in the Oswestry Disability Index during postoperative follow-up visits (*p* < 0.001). There were no major perioperative or long-term complications from the procedure in follow-up.

**Conclusion:**

The analysis of this cohort suggests successful long-term treatment of a diverse set of patients with PSPS who underwent spinal cord stimulation (SCS) and had meaningful improvement in quality of life and reduction in opioid consumption.

## Introduction

Spinal cord stimulation (SCS) in treatment of chronic, intractable truncal, and axial pain has been conducted for over 50 years (1). Improvement in stimulator technology is thought to have led to resurgence in use since 2005, when positive randomized control trials showed efficacy in treatment of patients with failed back surgery syndrome (FBSS) (2–4). Axial pain is nociceptive, or neuropathic pain is felt to be secondary to a spinal abnormality that does not have a radicular pattern and includes some forms of FBSS. Under the 2019 guidelines from the International Association for the Study of Pain, these patients are proposed to have “persistent spinal pain syndrome” (PSPS), which is subdivided into two types; type 1 (no previous spinal surgery) and type 2 (post-spinal surgery) (5). SCS is also indicated for other forms of back and lower limb radicular pain, such as failed neck surgery, complex regional pain syndrome (CRPS), and other poorly defined or unexplainable spinal pain syndromes (6). The incidence of patients who will develop PSPS after lumbar spinal surgery is variable and estimated between 10 and 40% with significant severe pain leading to some significant impact on quality of life (7). The mechanism of PSPS is a complex interaction of surgical factors (surgical complications, namely, hardware failure, infection, and hematoma) and other predisposing factors (psychosocial and anatomical factors). This can lead to exacerbation of preexisting spinal pathology causing nociceptive or neuropathic pain (5).

Medical therapy remains the most common form of treatment for PSPS. However, outcomes with existing pharmacological treatments are poor, with only partial relief of pain in 40–60% of patients (8). In addition, an increase in opioid prescription along with other psychosocial factors showed poor pain control and lead to higher addiction rates and was considered as a crisis in 2018 according to the Center of Disease Control (CDC) (9). Spinal cord stimulation devices may be employed as part of PSPS non-pharmacological management, which may lead to reduction in the use of potentially harmful treatments such as opioid analgesics.

In our study, we describe a retrospective case series of patients with type 1 PSPS and 2 with axial pain not associated to radicular pain or other pain syndromes like CRPS and persistent radicular pain post spinal surgery and treated with spinal cord stimulation, for which we reviewed postoperative outcomes including the use of prescription opioid analgesics, pain score, and disability score.

## Methods

### Institutional Case Series

A total of 25 patients had a trial of spinal cord implantation for axial neck and back pain in our institution between September 2017 and March 2021. The implant trial was conducted by the same senior neurosurgeon, under general anesthesia, and neuromonitoring. Since the coverage was under the universal health system, no further approval was needed before permanent implant once the trial was deemed successful, and to decrease surgical cost, permanent epidural leads were used. Leads were wrapped in subcutaneous pocket and an external connection to the external pulse generator was used. All incisions were closed and covered by a compressive dressing to minimize the risk of infection. Trials were deemed successful in case of an improvement in pain score by >50%. A decrease in pain medication consumption, in addition to improvement in daily activities and sleep, was also taken into consideration. Failure was not only defined by <50% improvement in pain score on VAS, but also by an increase or initiation of opioid consumption. Patient consent was obtained to complete a retrospective chart review. These patients were referred for persistent chronic neck and back pain lasting more than 6 months despite previous spinal surgical status and were refractory to conservative management and deemed ineligible for primary or revision spinal surgery by the specialized team.

Potential candidates for SCS device implantation underwent a strict multidisciplinary evaluation including medical and psychological assessments. Our multidisciplinary team consisted of an anesthesia pain physician who works closely with a neuropsychologist, a physiotherapist, a pharmacist, and a pain clinic nurse. All the patients were referred by their family physician for persistent chronic pain lasting more than 6 months despite previous spinal surgical status and were refractory to conservative management and deemed ineligible for primary or revision spinal surgery by the specialized team. Candidates were reviewed on a case-by-case basis in a multidisciplinary meeting that included an anesthesiologist pain physician, a functional neurosurgeon, a neuropsychologist, a pharmacist, and a pain nurse coordinator. An initial assessment was conducted by the pain clinic nurse with detailed medical and surgical history. Pain was evaluated using VAS and ODI questionnaires. Prescription was reviewed by the pain clinic pharmacist. The patients were then evaluated clinically, and axial imaging including MRI and x-ray of the whole spine were reviewed. If they were deemed to be candidate for neuromodulation, a neuropsychological evaluation was conducted. The evaluation looked for depression, anxiety, catastrophizing, and patient expectation. It also helped to eliminate any major underlying psychological and cognitive disorders. Once all evaluations were completed, patients considered fit for neuromodulation therapy were evaluated by the neurosurgeon to assess their operability and to discuss in detail the surgical risks and consent. Age was restricted to patients younger than 80 years old, and contraindications included coagulation disorders, systemic infection, decreased cognitive function, major medical comorbidities that contraindicate surgical intervention (unstable cardiac disease, active cancer, etc.), substance abuse disorder, psychological disorder (severe depression, bipolar disorder, and previous history of suicidal attempts and major psychosis disorder), and inability to manipulate the programmer. Patients who were eligible were offered a 7-to-14 day-trial with an external pulse generator.

### Procedure

After discussion, clear consent was obtained, and the patients were admitted for implantation. All the cases were conducted under general anesthesia in an operating room under strict sterile condition with neuromonitoring and imaging. The implant procedure was performed by the same senior functional neurosurgeon, and percutaneous permanent leads were favored but surgical leads were used in some cases because of anatomical deformities and previous complex spine surgery history. Since a permanent implant was used, a connection to an external extension was performed to facilitate internalization procedure once the trial is deemed successful. The patients were observed post-surgery overnight in a neurosurgical ward and discharged home the morning after for 7–14 days trial. Before discharge, a programming session was performed and two different programs were set, a tonic and a high frequency program (Burst, Abbott^®^ DTM, Medtronic^®^) after identification of good coverage of the painful area and initiation of comfortable level of stimulation. The patients also received education from the neuromodulation team on the programmer and how to navigate between programs.

### Trial and Follow-Up

During the trial period, the patients noted their pain level using VAS (0–10) at least 3 times daily, and occurrence of exacerbations was recorded. They also wrote down a diary of their pain medication consumption, daily activities, and sleep duration and quality. On trial days 3 and 6, a virtual follow-up was completed to decide among internalization, cessation, and a 1-week trial extension. The patients were discharged home on day one post epidural implantation because of the large area of coverage (distance from hospital can be as far as 450 km or 240 miles) by the single center covering two Atlantic provinces. Because of the distance and since trials are best evaluated in regular daily patient personal settings, virtual follow-up was adapted. This follow up lasted 15–30 min and conducted by the implanter and the neuromodulation nursing team where a review of the setting used was conducted, and all noted pain scores, activities, and medications were gathered.

Patients who had a successful trial were assigned for the internalization procedure. The same company provider for the external pulse generator is chosen for internalization. The procedure was performed under local-assisted anesthesia. Removal of the external extension and pulse generator was conducted, and an internal pulse generator was implanted to the left lower back region at the level of the supero-posterior iliac crest. The successful program from the trial was copied and verified clinically with the patients before discharge. The patients were followed after 2 weeks for wound evaluation and general check-up and then after 3, 6, and 12 months for the first year and on a biennial frequency thereafter. An evaluation with the Oswestry Disability Index (ODI), on the impact of lower back pain was completed in both pre-operative stage and 6 months postoperative follow-up (10).

Data were collected by chart review including electronic and patient-reported records of prescriptions from clinical encounters. Opioid prescription data for all the trialed patients were converted to morphine equivalents per day (Meq/D) using standard conversion tables (11). The data were described by mean and calculated standard error of the mean (SEM). Student's *t*-test was conducted for paired pre- and postoperative comparisons.

## Results

A total of 25 patients who had a spinal cord stimulation trial for persistent axial pain and absent radicular pain despite previous spinal surgical status in our institution between September 2017 and July 2021 were included. Demographics for this group are found in Table 1.

**Table 1:** Demographics and preoperative characteristics (*n* = 25).

Gender
Male	14 (56%)
Female	11 (44%)
Age (mean ± SEM)	58.2 [55.8–60.7]
Diagnosis
Low back pain without previous surgery	13 (52%)
Low back pain with previous surgery	7 (28%)
Chronic neck pain with previous surgery	3 (12%)
Chronic neck pain without previous surgery	2 (8%)
Preoperative analgesics
Opioids	11 (44.0%)
Gabapentinoids	10 (40.0%)
NSAIDs	9 (36.0%)
Antidepressants (TCAs, SNRIs)	7 (28.0%)

**Figure 1:**
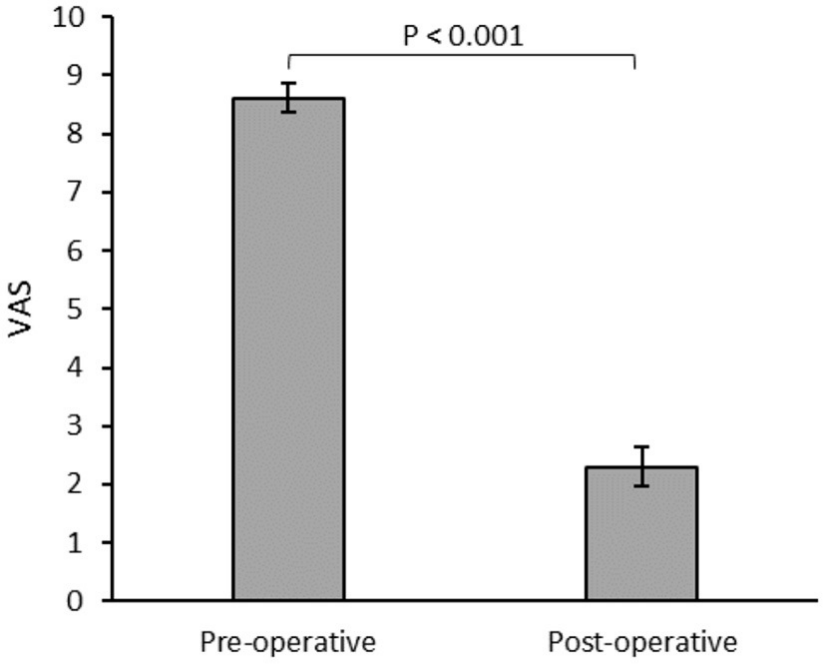
Mean visual analog scale (VAS) for patients (*n* = 23) assessed during the preoperative and 6 months postoperative follow-ups.

Postoperative mean pain score on the visual analog scale (VAS) was significantly lower in patients with internalized devices at 6 months (Figure 1). Two patients failed the initial trial period and did not undergo permanent device internalization. Of these, one reported worsened pain and poor tolerance to stimulation, and initiated opioid analgesics postoperatively. The other patient reported no change in baseline level and later during the same year developed a lumbar disc hernia necessitating a surgical intervention that improved their pain. There were no perioperative complications such as bleeding or infection.

**Figure 2:**
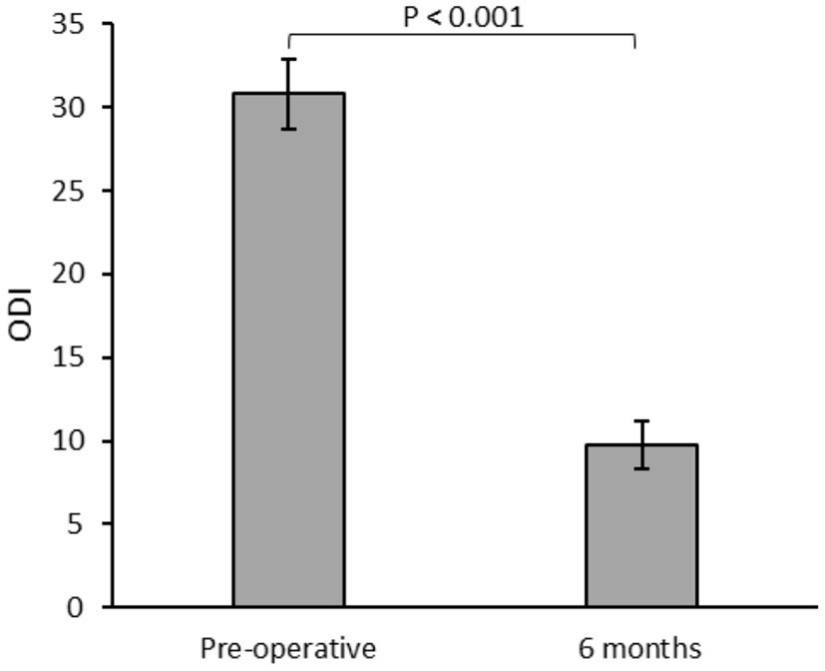
Mean Oswestry Disability Index (ODI) for patients (*n* = 23) assessed during the preoperative and 6 months postoperative follow-ups.

All implanted patients with successful trials had office follow-ups for a minimum of 6 months postoperatively, with a median of 29.8 months of follow-ups (6–40.7 months). When assessed in follow-ups, the patients showed a significant reduction in mean ODI (Figure 2).

### Opioids and Pain Management

**Figure 3:**
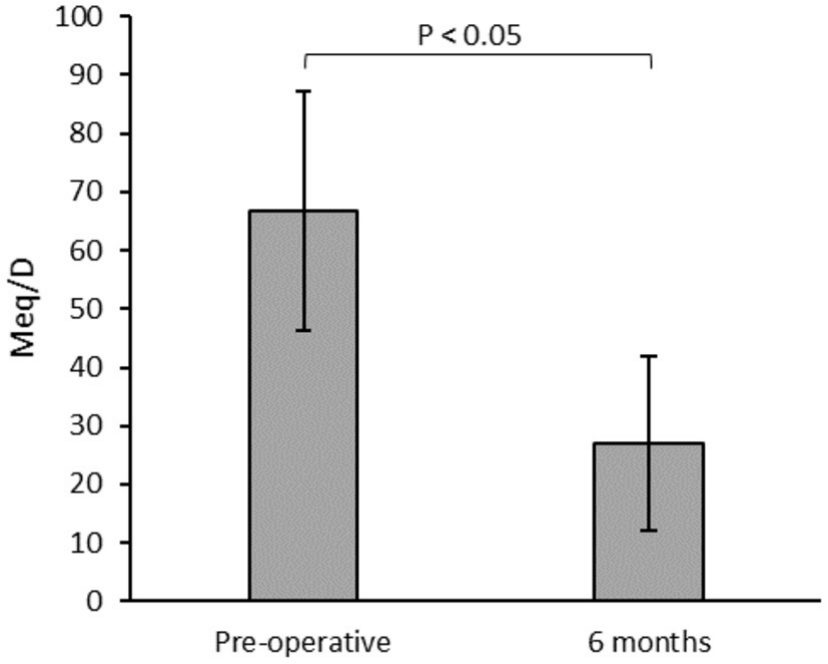
Mean morphine equivalents per day (Meq/D) for patients with pre-operative opioid consumption (*n* = 12) measured during the preoperative and 6 months postoperative follow-ups.

There were 12 patients who required preoperative opioid analgesics. No patients who were previously opioid-naïve initiated opioid therapy by the 6-month follow-up. Patients assessed at 6-month follow-up had a statistically significant reduction in mean morphine equivalents per day (Figure 3). Overall, 9 (75%) of the 12 patients with preoperative opioid therapy showed a decrease in consumption in the postoperative period. Six (50%) patients consuming pre-operative opioids showed complete cessation of all opioid analgesics after 6 months of follow-up. Of the remaining patients, 4 showed a reduction in the opioid dose by an average of 58.8%, and 2 had no change. No patients with internalized devices had an increase in opioid dose. One patient was on ketamine infusion every 2 weeks in their local emergency department for pain exacerbation and had 2 visits in an 18-month period, mainly after resuming a physically demanding job. Two other emergency visits for acute exacerbation were also noted in two different patients.

### Complications

Three patients developed battery failure where the generator reached its end of life and necessitated early revision. One patient died during the follow-up period because of other medical illnesses.

There was no infection observed in the first 6 months despite doing a permanent lead trial. One patient underwent device removal despite major benefit from the stimulation because of development of pulse generator pocket infection 19 months after the initial implantation due to a non-related traumatic injury with wound dehiscence.

The low risk of infection could be explained by the surgical closure of all wounds and compressive dressing. In addition, we consider the surgery as spinal clean-type with foreign body use, and prophylactic first generation cephalosporine is used 30 min before the procedure started and was continued for the first 24 h of patient hospitalization.

## Discussion

We report a retrospective case series of 25 patients with intractable chronic axial pain without radicular pain treated with spinal cord stimulation. Despite a variable pain etiology, we note improved outcomes following the implantation of a spinal cord stimulator with minimal adverse events including improved patient-reported pain outcomes, reduction in post-surgical opioid consumption, and lack of emergency department visit for pain exacerbations.

This has led us to evaluate the benefits of spinal cord stimulation for pain control, which are mostly centered on outcomes reported by the patients and are detected by reductions in their opioid consumption. The reduction in average opioid dose in our study was 37.9 Meq/D, consistent with a 59.4% reduction compared to preoperative doses and similar to previously reported ranges (42.4–64%) (12, 13). We note that SCS trial success was improved by strict selection criteria including preoperative multidisciplinary pain clinic assessment and psychological assessment, which has been previously described (13). This is particularly important in Canada, which has the second highest rate per capita of opioid prescription in the world with an increased prevalence of use, and current guidelines recommend specific criteria for initiation of opioids for chronic non-cancer pain (14).

In 2019, a systematic review concerning the use of neurostimulation devices for treatment of intractable chronic pain conditions affecting the spine/limbs found that, compared to medical therapy, spinal cord stimulation was significantly more likely to relieve pain particularly with newer technology (15). Some of these studies have specifically shown an opioid sparing effect of spinal cord stimulators for treatment of neuropathic pain (16). More recent publications have shown the efficacy of neurostimulation in treating persistent back pain prior to any spinal surgery irrespective of the presence of radicular symptoms (17, 18). Broader use of neurostimulation may lead to improved patient outcomes including reduction of risks associated with medical therapy like opioid dependence. It was also shown that improved pain helps ameliorate daily activities and overall quality of life.

In fact, we noted in our study that over their period of follow-up, which can be, in some cases, >3 years, the patients continued to show a positive impact on quality of life as evidenced by very high reduction in ODI scores.

It is important to highlight that advantages of well-controlled pain not only include improvement in the quality of life but also other clinical benefits. In fact, patients who report poorly managed chronic pain may be at a significantly increased risk of cardiovascular diseases (19). Moreover, a review of limited economic evaluations of spinal cord stimulation found that high initial costs are offset by long-term economic benefits particularly in failed back surgery syndrome and CRPS (20). We find that our patient cohort showed relative paucity of postoperative emergency department visits for exacerbations of their pain syndromes.

There were 2 patients who failed the initial device trial, giving a failure rate of 8.3%. This is similar to the 7.3% of patients with chronic pain of the trunk or limbs who failed the initial trial period in a recent study (17). From a safety perspective, only one patient had a delayed onset of infected hardware secondary to trauma, and there were no major adverse events such as hematoma or acute post-surgical infection.

## Limitations

This was a retrospective case series with no control group for comparison, and we could not examine our outcomes to control for meaningful confounding factors. Our observations were limited by the small sample size of our cohort due to budget restriction by the universal healthcare system, and reduced number of cases in the last 2 years due to coronavirus-2019 (COVID-19) pandemic restrictions and inability to operate elective cases.

## Conclusion

Spinal cord stimulation (SCS) has been widely studied, and different indications have emerged over the years. Our review redemonstrates the effectiveness of spinal cord stimulation in treating pain in a varied group of patients with type 1 and type 2 PSPS. This supports the use of spinal cord stimulation as a useful opioid-sparing treatment for management of patients with PSPS during the opioid crisis era. It also shows the importance of pain control with opioid reduction in improving the overall QOL and possible return to work of young active patients previously disabled by their pain. This needs to be investigated more from a cost effective perspective in order to have the therapy more available for patients especially in the universal healthcare system.

## Data Availability Statement

The raw data supporting the conclusions of this article will be made available by the authors, without undue reservation.

## Author Contributions

GM and JB prepared the manuscript. AS reviewed and edited. AE did senior editing and provided raw data from patient charts. All authors contributed to the article and approved the submitted version.

## Conflict of Interest

The authors declare that the research was conducted in the absence of any commercial or financial relationships that could be construed as a potential conflict of interest.

## Publisher's Note

All claims expressed in this article are solely those of the authors and do not necessarily represent those of their affiliated organizations, or those of the publisher, the editors and the reviewers. Any product that may be evaluated in this article, or claim that may be made by its manufacturer, is not guaranteed or endorsed by the publisher.
